# Probiotics to prevent necrotizing enterocolitis in very low birth weight infants: A network meta-analysis

**DOI:** 10.3389/fped.2023.1095368

**Published:** 2023-03-06

**Authors:** Ke-Zhao Zhou, Kang Wu, Lin-Xuan Deng, Man Hu, Yu-Xiang Luo, Li-Yan Zhang

**Affiliations:** Department of Neonatology, Fuzhou Children's Hospital of Fujian Medical University, Fuzhou, People's Republic of China

**Keywords:** necrotizing enterocolitis, very low birth weight infants, probiotics, network meta-analysis, prevention

## Abstract

**Objective:**

This study aims to review the evidence for the optimal regimen of probiotics for the prevention of necrotizing enterocolitis (NEC) in very low birth weight infants.

**Design:**

Through searching PubMed, EMBASE, Cochrane Library, and Web of Science till September 30, 2022, only randomized controlled trials were included to evaluate the optimal regimen of probiotics for the prevention of NEC in very low birth weight infants. The methodological quality of the included studies was assessed by the Cochrane risk of bias assessment tool (RoB 2), and the collected data were analyzed accordingly using Stata software.

**Results:**

Twenty-seven RCTs were included, and the total sample size used in the study was 529. The results of the network meta-analysis showed that Bovine lactoferrin + Lactobacillus rhamnosus GG (RR 0.03; 95% CI 0.00–0.35), Lactobacillus rhamnosus + Lactobacillus plantarum + Lactobacillus casei + Bifidobacterium lactis (RR 0.06; 95% CI 0.00–0.70), Bifidobacterium lactis + inulin (RR 0.16; 95% CI 0.03–0.91) were superior to the control group (Bifidobacterium lactis + Bifidobacterium longum) in reducing the incidence of NEC. The reduction in the incidence of NEC were as follows: Bovine lactoferrin + Lactobacillus rhamnosus GG (SUCRA 95.7%) > Lactobacillus rhamnosus + Lactobacillus plantarum + Lactobacillus casei + Bifidobacterium lactis (SUCRA 89.4%) > Bifidobacterium lactis + inulin (SUCRA 77.8%).

**Conclusions:**

This network meta-analysis suggests that Lactobacillus rhamnosus GG combined with bovine lactoferrin maybe the most recommended regimen for the prevention of NEC in very low birth weight infants.

## 1. Introduction

Necrotizing enterocolitis (NEC) is a gastrointestinal disease that seriously threatens the life of newborns. Clinically the infant presents with feed intolerance, increased gastric aspirates, vomiting, blood in the stool which may progress to very severe illness including shock and perforation. It is a disease that has plagued neonatal care for a long time and is still relatively common in very low birth weight infants (1). NEC is associated with neurodevelopmental delays, growth retardation, intestinal strictures and adhesions, and short bowel syndrome with or without intestinal failure (2). The high incidence of NEC cannot be ignored, and according to large multi-center neonatal network databases in the United States and Canada (3–5), NEC may occur in 7 out of 100 very low birth weight infants over the decades. Despite overall improvement in survival of preterm infants, a recent review suggests that the mortality and prevalence of NEC in very low birth weight infants has barely changed (6).

NEC, once established, is challenging to stop and has limited and expensive treatments. Treatment methods for NEC include antibiotics, gastric decompression, parenteral nutrition, etc. (7). It is not clear whether NEC is a single entity or a combination of similar entities and while progress has been made in understanding the pathogenesis of NEC there is still lack of clarity on many fronts which has perhaps contributed to a lack of significant progress in the treatment of NEC over the last many decades (8). On the other hand, although very low birth weight infants account for only a small proportion of newborns, the cost of treatment is indeed disproportionate. It has been reported that NEC causes more than 1 billion dollars in losses to medical institutions (9). It is worth noting that about 40% of NEC cases require surgical intervention (10), and the cost of treatment for infants requiring surgery has also significantly increased. All these factors lead to a considerable economic burden on the family and society.

Multiple research studies have explored various interventions for prevention of NEC including the provision of human milk and microbial therapeutics, with probiotic therapy garnering the most attention. Shiloh R. Lueschow et al. found that Bifidobacterium infantis EVC001 can prevent NEC in mice through anti-inflammatory and epithelial barrier-restoring properties (11). The study by Xiu-Li Zhu et al. has shown that probiotics supplementation can reduce the incidence and severity of NEC in preterm neonates (12), which seems to be related to the functions of probiotics in regulating immunity and inhibiting the imbalance of intestinal flora.

Most studies in the past decade have suggested that probiotics can significantly reduce the risk of NEC; However, it is unclear which probiotic or combination of probiotics is more effective (13) and what the optimal dose is. Moreover data on VLBW are scarce, and other related studies have not reported on specific strains of probiotics (14). Therefore, this study aimed to compare the effects of various probiotic regimens on NEC through a network meta-analysis, with direct or indirect comparisons, and to estimate the rank order of each combination. This hopefully will help target further research as well as facilitate improvements in practice.

## 2. Materials and methods

### 2.1. Search strategy

This network meta-analysis was conducted following the PRISMA statement, and the protocol for this study was registered in the International Platform of Registered Systematic Review and Meta-analysis Protocols (number INPLASY2022110001).

The researchers searched PubMed, EMBASE, Cochrane Library, and Web of Science till September 30, 2022. The search strategy was constructed around the PICOS tool: (*P*) Population: very low birth weight infants; (I) Intervention: probiotics; (C) Comparator: control group with only placebo or another probiotic usage; (O) Outcomes: necrotizing enterocolitis. (S) Study type: RCTs. The detailed search strategy is shown in (Table 1) (PubMed is used as an example).

**Table 1:** Search strategy on PubMed.

#1	Enterocolitis Necrotizing [MeSH Terms]
#2	Necrotizing Enterocolitis [Title/Abstract]
#3	#1 OR #2
#4	Probiotics [MeSH Terms]
#5	Probiotic [Title/Abstract]
#6	#4 OR #5
#7	randomized controlled trials [Publication Type]
#8	#3 AND #6 AND #7

### 2.2. Inclusion criteria

(1) Study designed as RCT; (2) Neonates with birth weight <1500 g; (3) Interventions included probiotics; (4) Reported outcomes included NEC stage ≥ II (Bell staging criteria); (5) The incidence of outcomes given by the study.

### 2.3. Exclusion criteria

(1) Studies from non-randomized controlled trials, including quasi-randomized controlled trials, non-human subjects, case reports, protocols, correspondence, or conference abstracts; (2) Studies with incomplete or unreported data.

### 2.4. Study selection

Literature was screened and excluded using the literature management software Endnote. Two researchers first screened papers by title to exclude duplication, non-randomized controlled trial studies, correspondence, review papers, and conference papers. Two researchers then read the abstracts to determine which studies to include and exclude. Finally, two researchers performed full-text readings to further identify the included literature. During this process, two researchers independently screened the literature and compared the remaining literature to determine whether they could be included in the study. Any conflicts were resolved by discussion with a third author.

### 2.5. Data extraction

A nine-item, standardized, and pre-selected data extraction form was used to record data from included studies under the following headings: (1) author, (2) year of publication, (3) country, (4) sample size, (5) mortality, (6) number of people progressing to NEC, (7) mean age, (8) details of the intervention, (9) overall risk of bias.

### 2.6. Risk of bias in individual studies

Two researchers independently assessed the risk of bias (RoB 2) according to the Cochrane Handbook version 6.1.0 tool for assessing RoB 2 in RCTs. Five items were considered: (1) randomization process, (2) deviations from intended interventions, (3) missing outcome date, (4) measurement of the outcome, and (5) selection of the reported result. The risk of bias for each domain can be classified into three levels: low risk, some concerns and high risk. If the risk of bias assessment for all domains is “low risk”, then the overall risk of bias is “low risk”; If the risk of bias assessment in some domains are “some concerns” and there is no “high risk” domain, then the overall risk of bias is “some concerns”; As long as the risk of bias assessment in one domain is “high risk”, the overall risk of bias is “high risk”.

### 2.7. Data analysis

In studies using probiotics as an intervention, outcome variables were dichotomized and expressed as risk ratios (RR) and 95% confidence intervals (CI). Due to potential differences between studies, we decided to use a random-effects model rather than a fixed-effects model to analyze the data.

Data were compiled and analyzed using Markov chain Monte Carlo simulation chains of Stata software (version 15.1) based on a Bayesian framework according to the PRISMA NMA instruction manual. To quantify and demonstrate the agreement between indirect and direct comparisons, we used the nodal method calculated according to the instructions in Stata. The consistency test was passed if the *P*-value was > 0.05.

We presented and described network diagrams for different probiotic usage using Stata software. In the presented network diagrams, each node represents a different probiotic usage, and the lines connecting the nodes represent a direct comparison between interventions. The size of each node and the width of the connecting lines are proportional to the number of trials.

The evaluation of the intervention was summarized and presented in the form of a *P* score. The *P* score was considered as a frequency analog to surface under the cumulative ranking curve (SUCRA) values, a measure of the degree of certainty that one treatment is superior to another. The *P* score ranges from 0 to 1, with 1 representing the best treatment without uncertainty and 0 the worst treatment without uncertainty. The P score or SUCRA could be effectively expressed as a percentage of intervention effectiveness or acceptability, but this score should be interpreted with caution unless there is a genuine clinically meaningful difference between interventions. Small-scale studies could lead to publication bias in NMA, for which we created network funnel plots and checked them visually using symmetry criteria.

## 3. Results

### 3.1. Study and identification and selection

**Figure 1:**
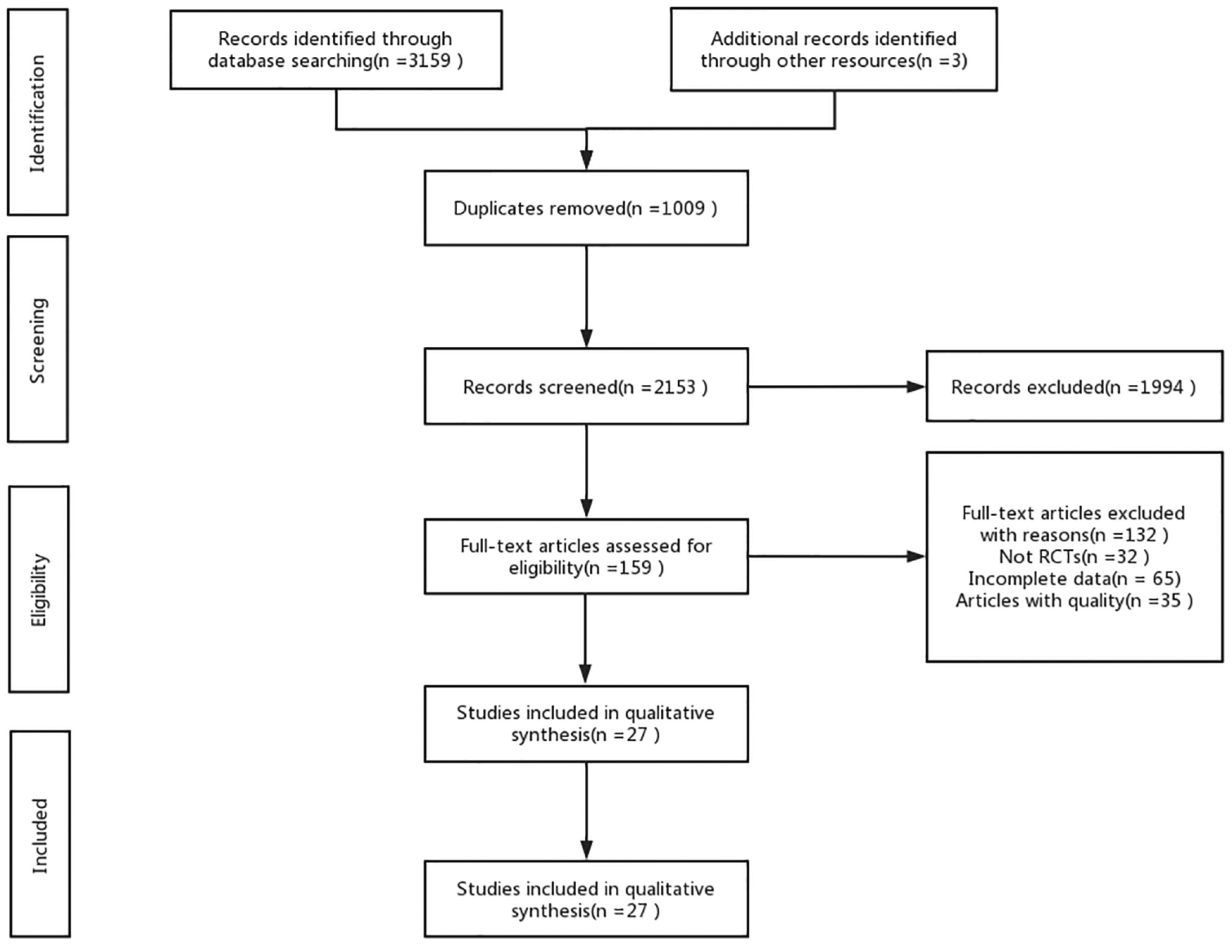
Flow diagram of literature selection.

A total of 3,159 documents were retrieved from the electronic database, and an additional three documents were manually searched. After eliminating duplicates, the remaining 2,153 documents were read for titles and abstracts, and 1,994 documents were again excluded. The remaining 159 documents were read in full, and 132 documents were again excluded (for reasons including non-randomized controlled trials, incomplete data, conference papers, and failure to meet the interventions included in this review), leaving a final remaining 27 documents to be included in this study. (Figure 1).

### 3.2. Quality assessment of the included studies

Seventeen studies were defined as low risk, eight as some concerns, and two as high risk. Only three of these studies did not achieve simultaneous blinding of subjects and measures. Specific details are presented in (Supplementary Table S1).

### 3.3. Characteristics of the included studies

In total, we included studies from 27 randomized controlled trials, which included 529 patients diagnosed with NEC. Interventions included Bovine lactoferrin + Lactobacillus rhamnosus GG (2 studies) (15, 16), Bovine lactoferrin (2 studies) (15, 16), Lactobacillus rhamnosus + Lactobacillus planvtarum + Lactobacillus casei + Bifidobacterium lactis (2 studies) (17, 18), Bifidobacterium lactis + inulin (1 study) (19), inulin (1 study) (19), Bifidobacterium lactis + Bifidobacterium longum (1 study) (20), Bifidobacterium lactis (3 studies) (19–21), Bifidobacterium longum (1 study) (20), Bifidobacterium bifidum + Lactobacillus acidophilus (2 studies) (22, 23), Lactobacillus rhamnosus (3 studies) (24–26), Bifidobacterium infantis + Streptococcus thermophilus + Bifidobacterium lactis (2 studies) (27, 28), Lactobacillus sporogenes (1 study) (29), Lactobacillus reuteri DSM 17938 (4 studies) (30–33), Lactobacillus rhamnosus GG + Bifidobacterium infantis (2 studies) (34, 35), Saccharomyces boulardii (2 studies) (36, 37), Bifidobacterium breve + Bifidobacterium infantis + Bifidobacterium longum (1 study) (38) and Bifidobacterium breve (4 studies) (38–41). There were two studies from Asia, three studies from America, eighteen studies from Europe, and four studies from Oceania. The characteristics of the included studies are shown in (Table 2).

**Table 2:** Characteristics of the studies included in the meta-analysis.

Author	Year	Country	Sample		Mortality		Progression of NEC		Age.mean (SD)		Interventions		Interventions frequency	Overall Bias
			T	C	T	C	T	C	T	C	T	C		
Paolo Manzoni1	2014	Italy	①247	258	①5	18	①5	14	①29.7 (2.5)	29.4 (3.1)	①BLF	Placebo	Once a day	Low risk
			②238		②9		②0		②29.6 (2.8)		②BLF and LGG			
Gamze Demirel	2013	Turkey	135	136	5	6	6	7	29.4 (2.3)	29.2 (2.5)	S.b	Placebo	Once a day	Some concerns
Ozge Serce	2013	Turkey	104	104	5	4	7	7	28.8 (2.2)	28.7 (-2.1)	S.b	Placebo	Twice a day	Low risk
Kate Costeloe1	2015	UK	650	660	54	56	61	66	30.6 (6.5)	30.9 (6.6)	B.b	Placebo	Once a day	Low risk
Sanjay Patole	2014	Australia	79	80	0	0	0	1	29 (26-30; 23-32)	28 (26-29; 23-33)	B.b	Placebo	Once a day	Some concerns
Hung-Chih Lin	2014	China	217	217	4	20	4	14	/	/	B.bi and L.a	Placebo	Twice a day	Low risk
Mehmet Yekta Oncel	2014	Turkey	200	200	20	27	8	10	28.2 (2.4)	27.9 (2.5)	L.r	Placebo	Once a day	Low risk
Susan E. Jacobs	2013	Australia	548	551	27	28	4	11	27.9 (2.0)	27.8 (2.0)	B.i S.t and B.l	Placebo	Once a day	Low risk
Stephane Hays	2015	France	①50	52	1	1	①2	3	29.0 (28.1; 30.1)	29.4 (27.9; 30.6)	①B.l	Placebo	Once a day	Low risk
			②48				②1				②B.lo			
			③47				③5				③B.l and B.lo			
Dilek Dilli	2015	Turkey	①100	100	①3	12	①2	18	①28.8 (1.9)	28.2 (2.2)	①B.l	Placebo	Once a day	Low risk
			②100		②2		②12		②29.0 (1.7)		②inulin			
			③100		③3		③4		③28.9 (1.9)		③B.l and inulin			
Kate Costeloe2	2016	UK	611	619	54	56	57	62	/	/	B.b	Placebo	Once a day	High risk
İpek Güney-Varal	2017	Turkey	70	40	1	9	0	4	29.7 (1.9)	29.3 (1.7)	LGG, L.p, L.c and B.l	Placebo	Once a day	Low risk
M Al-Hosni	2012	USA	50	51	3	4	2	2	25.7 (1.4)	25.7 (1.4)	LGG and B.i	Placebo	Once a day	Some concerns
Ozge Serce Pehlevan	2019	Turkey	104	104	6	3	0	4	29 (1.9)	28 (2.2)	LGG, L.p, L.c and B.l	Placebo	Once a day	Low risk
W.A. Mihatsch	2009	Germany	93	90	2	1	2	4	26.6 (1.8)	26.7 (1.7)	B.l	Placebo	Once a day	Some concerns
Iwona Sadowska -Krawczenko	2012	Poland	30	25	1	0	1	4	29 (27-31)	30 (27-31)	LGG	Placebo	Twice a day	Low risk
Varaporn Saengtawesin	2014	Thailand	31	29	0	0	1	1	31.0 (1.82)	30.59 (1.76)	B.bi and L.a	Placebo	Twice a day	Some concerns
Carlo Dani	2002	Italy	295	290	0	2	4	8	30.8 (2.4)	30.7 (2.3)	LGG	Placebo	Once a day	Low risk
FN Sari	2011	Turkey	110	111	3	4	6	10	29.5 (2.4)	29.7 (2.4)	L.s	Placebo	Once a day	High risk
Paolo Manzoni2	2009	Italy	①153	168	①4	12	①3	10	①29.6 (2.5)	29.5 (3.2)	①BLF	Placebo	Once a day	Low risk
			②151		②6		②0		②29.8 (2.8)		②BLF and LGG			
Erica L. Plummer2	2021	Australia	229	230	/	/	11	18	28.6 (27.2-30)	28.1 (26.5-29.5)	B.i, S.t and B.l	Placebo	Once a day	Some concerns
T. Havranek	2013	USA	15	16	/	/	0	1	25.9 (1.3)	25.9 (1.5)	LGG and B.i	Placebo	Once a day	Low risk
Gayatri Athalye-Jape	2022	Australia	①86	29	①8	0	①3	0	①26.2 (24.4-27.2)	26.1 (25.2-26.9)	①B.b	Placebo	Once a day	Some concerns
			②87		②12		②3		②26.3 (24.7-27.1)		②B.b, B.i and B.lo			
P. Manzoni	2006	Italy	39	41	5	6	1	2	29.6 (5)	29.3 (4)	LGG	Placebo	Once a day	Low risk
JohanneE. Spreckels	2021	Sweden	48	57	0	0	2	5	25.4 (1.3)	25.8 (1.1)	L.r	Placebo	Once a day	Some concerns
Erik Wejryd	2019	Sweden	68	66	5	5	7	8	25.5 (1.2)	25.5 (1.3)	L.r	Placebo	Once a day	Low risk
Nancy Patricia	2015	Mexico	24	20	1	/	/	10	31.2 (2.39)	31.7 (2.47)	L.r	Placebo	Once a day	Low risk

Note: BLF and LGG, bovine lactoferrin and lactobacillus rhamnosus GG; LGG, L.p, L.c and B.l, Lactobacillus rhamnosus, lactobacillus plantarum, lactobacillus casei and bifidobacterium lactis; B.l and inulin, bifidobacterium lactis and inulin; B.l, bifidobacterium lactis; B.lo, bifidobacterium longum; B.bi and L.a, bifidobacterium bifidum and lactobacillus acidophilus; BLF, bovine lactoferrin; LGG, lactobacillus rhamnosus GG; B.i, S.t and B.l, bifidobacterium infantis, streptococcus thermophilus and bifidobacterium lactis; L.s, lactobacillus sporogenes; L.r, lactobacillus reuteri; LGG and B.i, lactobacillus rhamnosus GG and bifidobacterium infantis; S.b, saccharomyces boulardii; B.b, B.i and B.lo, bifidobacterium breve, bifidobacterium infantis and bifidobacterium longum; B.b, bifidobacterium breve; B.l and B.lo, bifidobacterium lactis and bifidobacterium longum.

**Table 3:** League table for outcome.

BLF + LGG	LGG + L.p + L.c + B.l	B.l + inulin	B.l	B.lo	B.bi + L.a	BLF	LGG	B.i + S.t + B.l	L.r	L.s	inulin	LGG + B.i	S.b	B.b + B.i + B.lo	B.b	placebo	B.l + B.lo
BLF + LGG	1.87 (0.10,33.60)	5.11 (0.52,50.66)	6.33 (0.69,58.44)	5.66 (0.29,111.78)	7.68 (0.80,73.74)	8.81 (1.11,69.94)	9.68 (1.04,90.11)	12.11 (1.48,99.40)	13.95 (1.71,114.00)	13.87 (1.44,133.23)	16.73 (1.95,143.67)	17.94 (1.29,249.60)	22.09 (2.57,189.72)	24.76 (2.05,299.33)	22.12 (2.93,166.97)	23.81 (3.21,176.71)	31.66 (2.83,354.63)
0.53 (0.03,9.61)	LGG + L.p + L.c + B.l	2.73 (0.26,28.96)	3.39 (0.34,33.48)	3.03 (0.15,62.94)	4.11 (0.40,42.19)	4.71 (0.51,43.36)	5.18 (0.52,51.60)	6.48 (0.73,57.15)	7.46 (0.85,65.55)	7.42 (0.72,76.22)	8.95 (0.97,82.48)	9.60 (0.65,141.49)	11.82 (1.28,108.91)	13.25 (1.03,170.23)	11.83 (1.45,96.27)	12.74 (1.59,101.95)	16.93 (1.42,202.05)
0.20 (0.02,1.94)	0.37 (0.03,3.87)	B.l + inulin	1.24 (0.31,5.00)	1.11 (0.09,12.92)	1.50 (0.33,6.94)	1.72 (0.44,6.70)	1.89 (0.43,8.35)	2.37 (0.65,8.58)	2.73 (0.76,9.81)	2.71 (0.59,12.54)	3.27 (1.02,10.52)	3.51 (0.46,26.96)	4.32 (1.11,16.83)	4.84 (0.76,30.92)	4.33 (1.38,13.60)	4.66 (1.53,14.20)	6.19 (1.10,34.75)
0.16 (0.02,1.46)	0.30 (0.03,2.92)	0.81 (0.20,3.26)	B.l	0.89 (0.09,8.47)	1.21 (0.29,5.02)	1.39 (0.41,4.78)	1.53 (0.39,6.02)	1.91 (0.60,6.07)	2.20 (0.70,6.94)	2.19 (0.53,9.08)	2.64 (0.84,8.31)	2.83 (0.40,20.08)	3.49 (1.01,12.01)	3.91 (0.67,22.84)	3.49 (1.29,9.45)	3.76 (1.44,9.82)	5.00 (1.22,20.54)
0.18 (0.01,3.49)	0.33 (0.02,6.87)	0.90 (0.08,10.55)	1.12 (0.12,10.61)	B.lo	1.36 (0.12,15.66)	1.56 (0.15,16.19)	1.71 (0.15,19.19)	2.14 (0.21,21.38)	2.47 (0.25,24.53)	2.45 (0.21,28.30)	2.96 (0.29,30.21)	3.17 (0.19,51.74)	3.90 (0.38,40.65)	4.38 (0.31,62.58)	3.91 (0.42,36.18)	4.21 (0.46,38.35)	5.60 (0.63,49.84)
0.13 (0.01,1.25)	0.24 (0.02,2.50)	0.67 (0.14,3.07)	0.82 (0.20,3.41)	0.74 (0.06,8.49)	B.bi + L.a	1.15 (0.31,4.22)	1.26 (0.30,5.28)	1.58 (0.46,5.38)	1.82 (0.54,6.16)	1.81 (0.41,7.95)	2.18 (0.59,8.03)	2.33 (0.32,17.30)	2.88 (0.78,10.60)	3.22 (0.53,19.76)	2.88 (0.98,8.47)	3.10 (1.09,8.83)	4.12 (0.75,22.73)
**0.11** **(0.01,0.90)**	0.21 (0.02,1.95)	0.58 (0.15,2.26)	0.72 (0.21,2.47)	0.64 (0.06,6.68)	0.87 (0.24,3.21)	BLF	1.10 (0.32,3.83)	1.38 (0.50,3.76)	1.58 (0.58,4.30)	1.58 (0.43,5.80)	1.90 (0.63,5.70)	2.04 (0.31,13.28)	2.51 (0.84,7.52)	2.81 (0.53,14.96)	2.51 (1.11,5.69)	2.70 (1.25,5.87)	3.59 (0.76,17.03)
**0.10** **(0.01,0.96)**	0.19 (0.02,1.92)	0.53 (0.12,2.33)	0.65 (0.17,2.57)	0.58 (0.05,6.55)	0.79 (0.19,3.33)	0.91 (0.26,3.17)	LGG	1.25 (0.39,4.03)	1.44 (0.45,4.61)	1.43 (0.34,6.01)	1.73 (0.49,6.03)	1.85 (0.26,13.25)	2.28 (0.65,7.96)	2.56 (0.43,15.09)	2.28 (0.83,6.29)	2.46 (0.92,6.54)	3.27 (0.62,17.30)
**0.08** **(0.01,0.68)**	0.15 (0.02,1.36)	0.42 (0.12,1.53)	0.52 (0.16,1.66)	0.47 (0.05,4.66)	0.63 (0.19,2.17)	0.73 (0.27,1.99)	0.80 (0.25,2.58)	B.i + S.t + B.l	1.15 (0.47,2.83)	1.15 (0.33,3.92)	1.38 (0.50,3.79)	1.48 (0.24,9.17)	1.82 (0.66,5.00)	2.04 (0.41,10.27)	1.83 (0.91,3.65)	1.97 (1.03,3.73)	2.61 (0.59,11.64)
**0.07** **(0.01,0.59)**	0.13 (0.02,1.18)	0.37 (0.10,1.32)	0.45 (0.14,1.43)	0.41 (0.04,4.04)	0.55 (0.16,1.87)	0.63 (0.23,1.71)	0.69 (0.22,2.22)	0.87 (0.35,2.13)	L.r	0.99 (0.29,3.38)	1.20 (0.44,3.26)	1.29 (0.21,7.93)	1.58 (0.58,4.31)	1.78 (0.36,8.87)	1.59 (0.80,3.13)	1.71 (0.91,3.20)	2.27 (0.51,10.05)
**0.07** **(0.01,0.69)**	0.13 (0.01,1.38)	0.37 (0.08,1.70)	0.46 (0.11,1.89)	0.41 (0.04,4.71)	0.55 (0.13,2.44)	0.63 (0.17,2.34)	0.70 (0.17,2.93)	0.87 (0.26,2.99)	1.01 (0.30,3.41)	L.s	1.21 (0.33,4.45)	1.29 (0.17,9.59)	1.59 (0.43,5.88)	1.79 (0.29,10.96)	1.59 (0.54,4.70)	1.72 (0.60,4.90)	2.28 (0.41,12.60)
**0.06** **(0.01,0.51)**	0.11 (0.01,1.03)	**0.31** **(0.10,0.98)**	0.38 (0.12,1.19)	0.34 (0.03,3.46)	0.46 (0.12,1.69)	0.53 (0.18,1.58)	0.58 (0.17,2.02)	0.72 (0.26,1.99)	0.83 (0.31,2.27)	0.83 (0.22,3.06)	inulin	1.07 (0.16,7.00)	1.32 (0.44,3.97)	1.48 (0.28,7.89)	1.32 (0.58,3.01)	1.42 (0.65,3.10)	1.89 (0.41,8.73)
**0.06** **(0.00,0.78)**	0.10 (0.01,1.54)	0.28 (0.04,2.19)	0.35 (0.05,2.50)	0.32 (0.02,5.15)	0.43 (0.06,3.17)	0.49 (0.08,3.20)	0.54 (0.08,3.86)	0.68 (0.11,4.18)	0.78 (0.13,4.80)	0.77 (0.10,5.73)	0.93 (0.14,6.09)	LGG + B.i	1.23 (0.19,8.04)	1.38 (0.14,13.23)	1.23 (0.22,6.93)	1.33 (0.24,7.32)	1.76 (0.20,15.55)
**0.05** **(0.01,0.39)**	**0.08** **(0.01,0.78)**	**0.23** **(0.06,0.90)**	**0.29** **(0.08,0.99)**	0.26 (0.02,2.67)	0.35 (0.09,1.28)	0.40 (0.13,1.20)	0.44 (0.13,1.53)	0.55 (0.20,1.50)	0.63 (0.23,1.72)	0.63 (0.17,2.32)	0.76 (0.25,2.28)	0.81 (0.12,5.30)	S.b	1.12 (0.21,5.97)	1.00 (0.44,2.28)	1.08 (0.49,2.35)	1.43 (0.30,6.80)
**0.04** **(0.00,0.49)**	**0.08** **(0.01,0.97)**	0.21 (0.03,1.32)	0.26 (0.04,1.49)	0.23 (0.02,3.27)	0.31 (0.05,1.90)	0.36 (0.07,1.89)	0.39 (0.07,2.31)	0.49 (0.10,2.46)	0.56 (0.11,2.81)	0.56 (0.09,3.44)	0.68 (0.13,3.60)	0.72 (0.08,6.94)	0.89 (0.17,4.75)	B.b + B.i + B.lo	0.89 (0.21,3.86)	0.96 (0.22,4.23)	1.28 (0.17,9.47)
**0.05** **(0.01,0.34)**	**0.08** **(0.01,0.69)**	**0.23** **(0.07,0.73)**	**0.29** **(0.11,0.77)**	0.26 (0.03,2.37)	0.35 (0.12,1.02)	**0.40** **(0.18,0.90)**	0.44 (0.16,1.21)	0.55 (0.27,1.10)	0.63 (0.32,1.25)	0.63 (0.21,1.85)	0.76 (0.33,1.72)	0.81 (0.14,4.56)	1.00 (0.44,2.27)	1.12 (0.26,4.84)	B.b	1.08 (0.83,1.40)	1.43 (0.36,5.66)
**0.04** **(0.01,0.31)**	**0.08** **(0.01,0.63)**	**0.21** **(0.07,0.65)**	**0.27** **(0.10,0.69)**	0.24 (0.03,2.17)	**0.32** **(0.11,0.92)**	**0.37** **(0.17,0.80)**	0.41 (0.15,1.08)	**0.51** **(0.27,0.97)**	0.59 (0.31,1.10)	0.58 (0.20,1.66)	0.70 (0.32,1.53)	0.75 (0.14,4.15)	0.93 (0.43,2.02)	1.04 (0.24,4.57)	0.93 (0.72,1.21)	placebo	1.33 (0.35,5.12)
**0.03** **(0.00,0.35)**	**0.06** **(0.00,0.70)**	**0.16** **(0.03,0.91)**	**0.20** **(0.05,0.82)**	0.18 (0.02,1.59)	0.24 (0.04,1.34)	0.28 (0.06,1.32)	0.31 (0.06,1.62)	0.38 (0.09,1.70)	0.44 (0.10,1.95)	0.44 (0.08,2.42)	0.53 (0.11,2.44)	0.57 (0.06,4.99)	0.70 (0.15,3.31)	0.78 (0.11,5.80)	0.70 (0.18,2.76)	0.75 (0.20,2.90)	B.l + B.lo

### 3.4. Network meta-analysis

**Figure 2:**
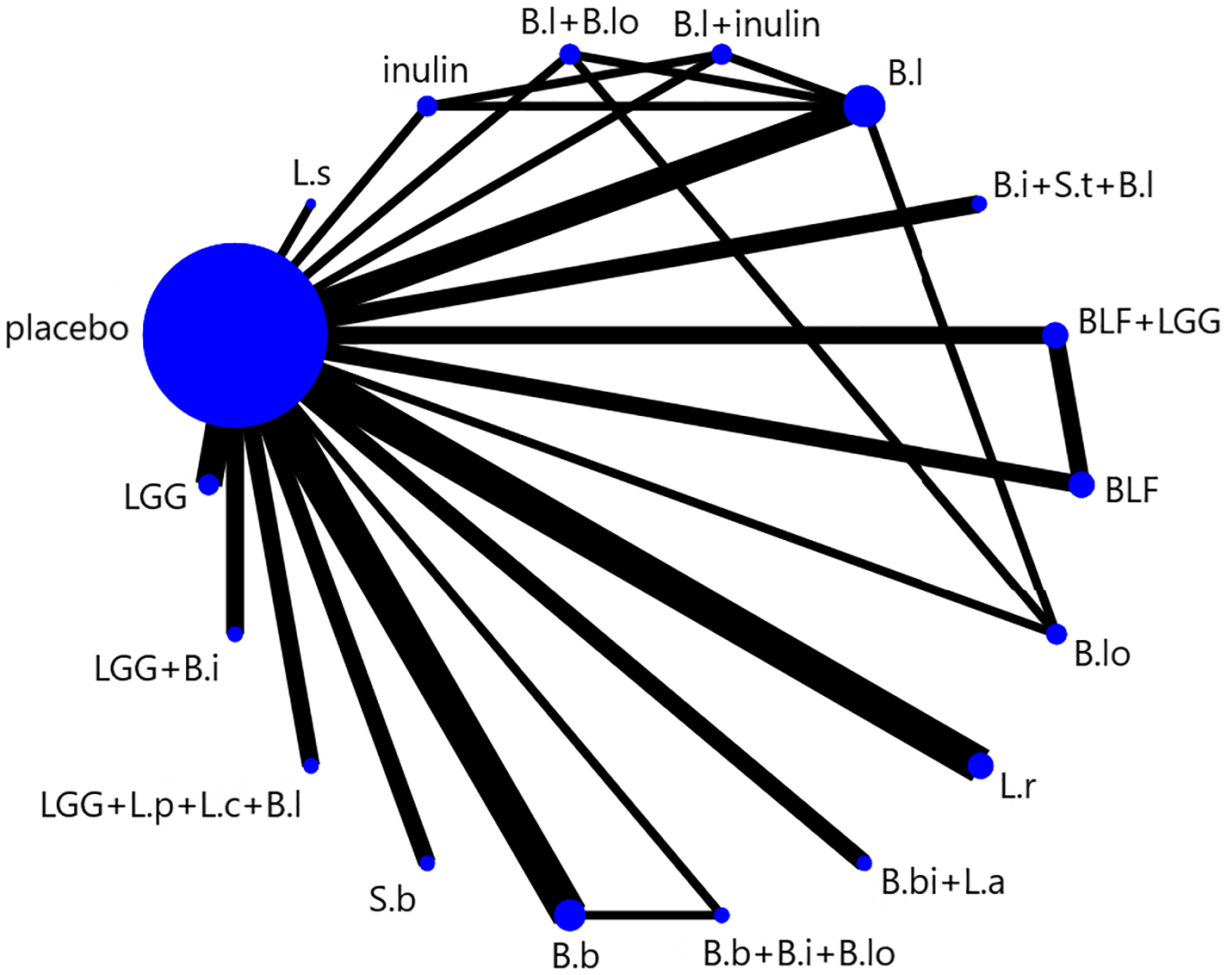
NMA figure.

The full NMA figure is shown in (Figure 2). All *P*-values for indirect and direct comparisons between all studies were tested for consistency and inconsistency, and most *P*-values were greater than 0.05, indicating that the effects of consistency between studies were acceptable. Details were shown in (Supplementary Table S2).

The results of the Network meta-analysis showed that Bovine lactoferrin + Lactobacillus rhamnosus (RR 0.03; 95% CI 0.00–0.35; Table 3), Lactobacillus rhamnosus + Lactobacillus plantarum + Lactobacillus casei + Bifidobacterium lactis (RR 0.06; 95% CI 0.00–0.70), Bifidobacterium lactis + inulin (RR 0.16; 95% CI 0.03–0.91), Bifidobacterium lactis (RR 0.20; 95% CI 0.05–0.82) were superior to the control group (Bifidobacterium lactis + Bifidobacterium longum) in reducing the incidence of NEC. Relative to the control group (placebo), Bifidobacterium bifidum + Lactobacillus acidophilus (RR 0.32; 95% CI 0.11–0.92) and Bifidobacterium infantis + Streptococcus thermophilus + Bifidobacterium lactis (RR 0.51; 95% CI 0.27–0.97) were superior to the control group (placebo) in reducing the incidence of NEC.

**Figure 3:**
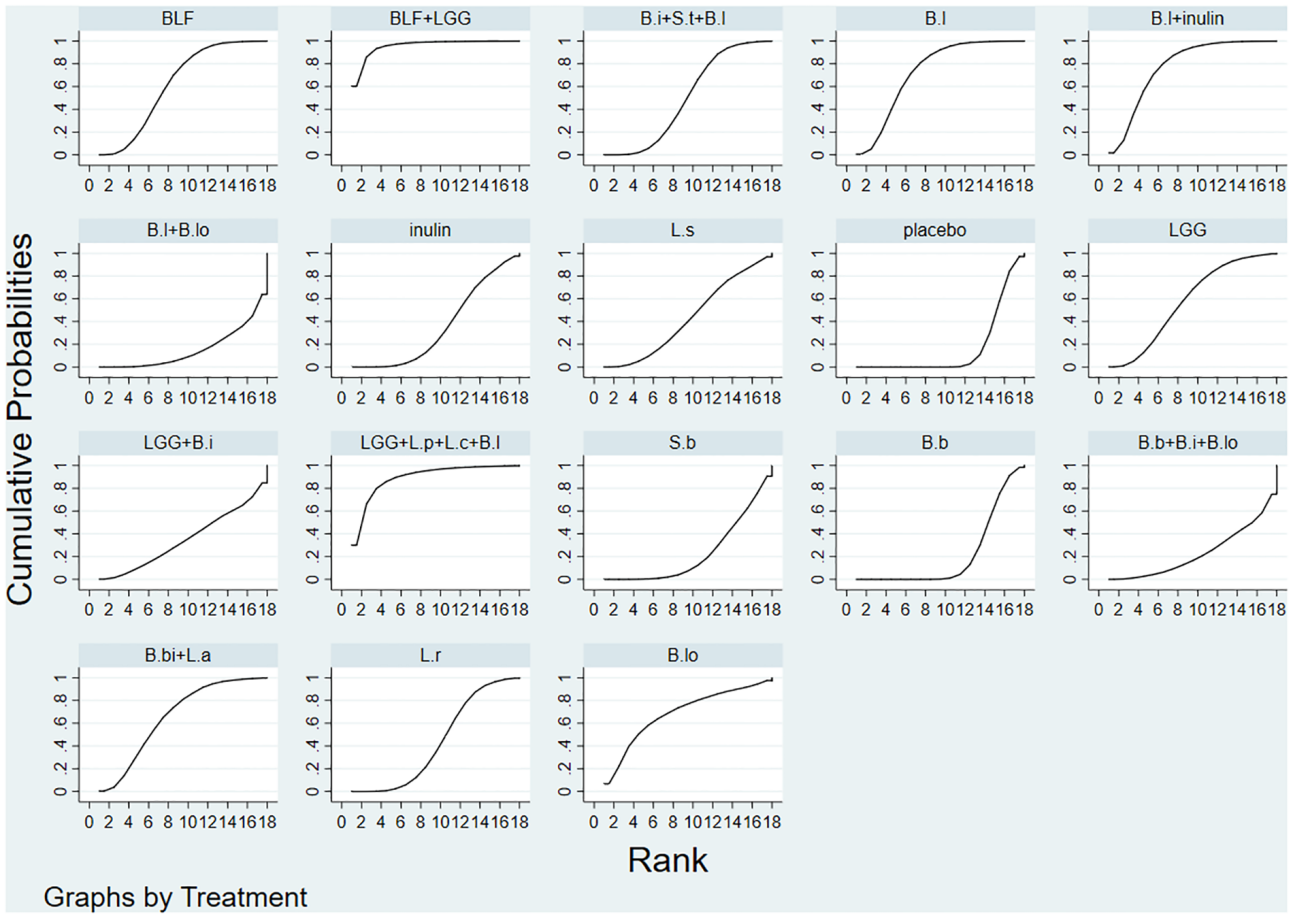
SUCRA plot.

Bayesian Markov chain Monte Carlo modeling revealed that Bovine lactoferrin + Lactobacillus rhamnosus had the highest probability of having the lowest rate of NEC (SUCRA 95.7%; Figure 3), followed by Lactobacillus rhamnosus + Lactobacillus plantarum + Lactobacillus casei + Bifidobacterium lactis (SUCRA 89.4%), and Bifidobacterium lactis + inulin (SUCRA 77.8%).

### 3.5. Publication bias test

**Figure 4:**
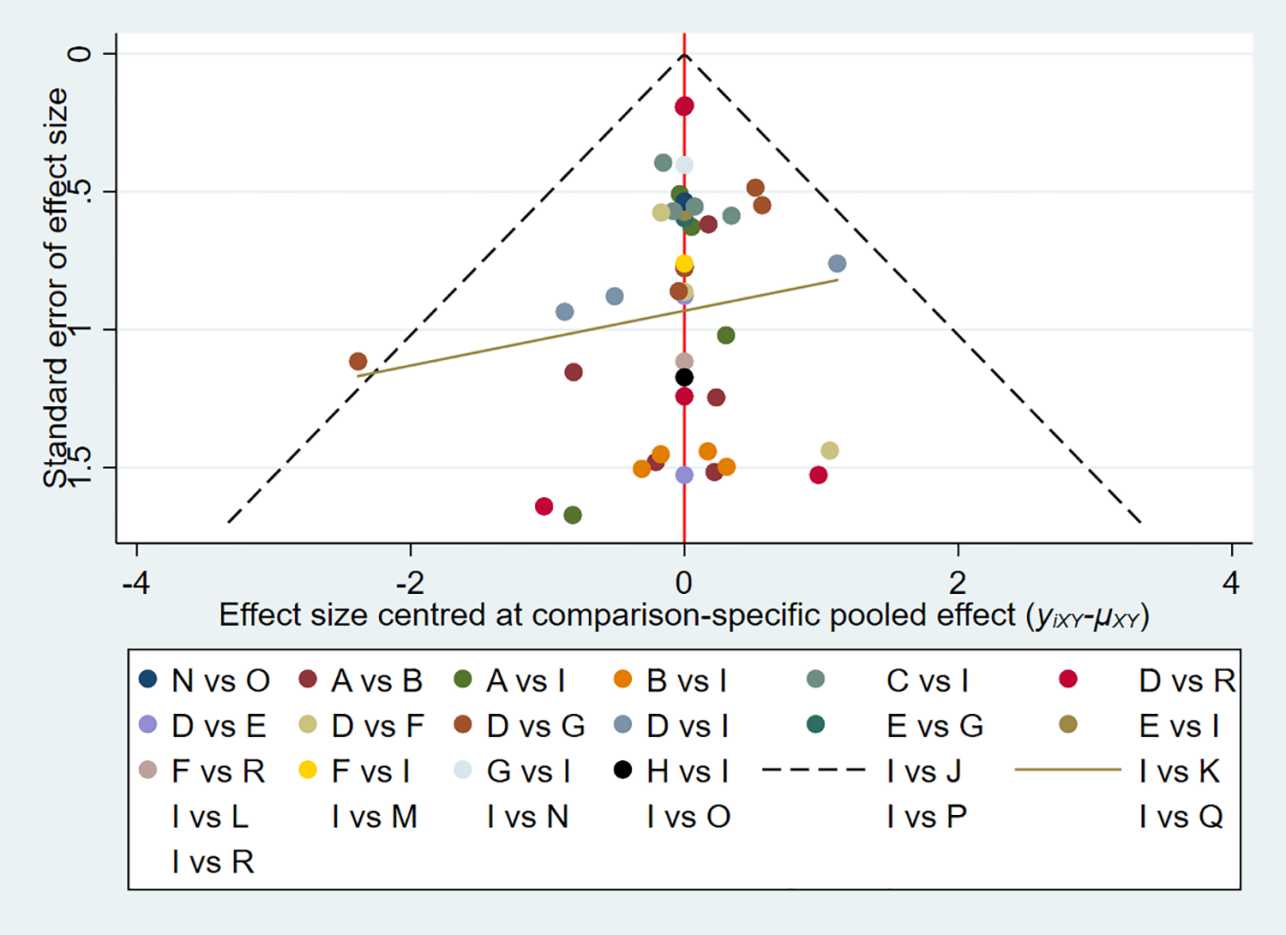
Funnel plot on publication bias.

We constructed separate funnel plots for all outcome indicators to test for possible publication bias. Visual inspection of the funnel plots did not reveal any significant publication bias (42). Details were shown in (Figure 4).

## 4. Discussion

NEC is a gastrointestinal disorder that has plagued the field of neonatology for a long time. Considering the morbidity and mortality of NEC, as well as the high cost of treatment and socioeconomic loss, it is important to prioritize research on NEC prevention and treatment. This study included 27 trials with 18 interventions, including 9,501 very low birth weight infants. We aimed to investigate which probiotics effectively prevent NEC in very low birth weight infants. This network meta-analysis concluded that Lactobacillus rhamnosus plus bovine lactoferrin might be the most appropriate regimen for preventing NEC in very low birth weight infants compared to a placebo or other probiotic control group.

Lactobacillus rhamnosus GG belongs to the genus Lactobacillus, a naturally occurring gram-positive bacterium that was originally isolated from the healthy human intestine (43). Lactobacillus rhamnosus GG has strong adhesion to intestinal cells and can also exert its high immune activity in the acidic pH environment of the digestive tract (44), which are prerequisites for colonization in the human intestine. Lactoferrin is a transferrin-like protein with anti-infective and anti-inflammatory properties (45) and is found in high levels in human colostrum and low levels in breast milk, tears, saliva, and semen. Lactoferrin can be processed from bovine or human milk, with GM rice and GM corn currently under study as promising new sources (46). Since bovine lactoferrin is cheaper than human lactoferrin, it is more readily used.

The studies by Paolo Manzoni et al. (15, 16) have shown that bovine lactoferrin combined with Lactobacillus rhamnosus GG can significantly reduce the incidence of NEC in very low birth weight infants, which is consistent with the results of this network meta-analysis. They believe that this might be related to the ability of lactoferrin to provide some anti-infection, nutrition, and immune regulation activity in the intestine to synergize with the effect of Lactobacillus rhamnosus GG against NEC in premature infants. These findings are also supported by a retrospective cohort study by Michael P. Meyer et al., who also showed that the cost of prevention was significantly lower than the cost of treatment (47). Lactobacillus rhamnosus GG can adhere to the intestinal epithelium and generate biofilms and attenuate the pro-inflammatory effects of cytokines and protect the mucosal barrier (48, 49). In addition, Lactobacillus rhamnosus GG can play an anti-pathogen role by stimulating non-specific immunity, increasing the secretion of interleukin-6 and expressing over 90 antimicrobial or immunomodulatory proteins (43, 48–50). Bovine lactoferrin may provide a broad-spectrum anti-pathogen effect by directly lysing microbial cell membranes (51, 52). Moreover, lactoferrin can also protect intestinal epithelial cells by down-regulating the highly expressed pro-inflammatory cytokines in intestinal epithelial cells, inhibiting the activity of free radicals and reducing the levels of oxidative products (53, 54).

It is obvious that Lactobacillus rhamnosus GG and bovine lactoferrin have the similar effects and create good conditions for the growth of beneficial bacteria, and can also inhibit the colonization of pathogens. The combined use can enhance the overall effect (55). The study by Po-Wen Chen et al. has shown that when the growth of probiotics is not optimum, bovine lactoferrin provides a more substantial prebiotic effect and promotes the growth of probiotics, including Lactobacillus rhamnosus GG (56).

A Position Paper by the European Society for Pediatric Gastroenterology Hepatology and Nutrition Committee on Nutrition and the European Society for Pediatric Gastroenterology Hepatology and Nutrition Working Group for Probiotics and Prebiotics indicates that the question of which probiotic strain or combination to use is mainly based on known literature (mainly case series and author's expertise) (57), these recommendations are based on very low certainty of evidence. Compared with other studies, this study compared the effects of various probiotic regimens on NEC through network meta-analysis, and obtained the optimal probiotic regimen by ranking each intervention. In addition, this study also analyzed specific strains of probiotics. In the studies included in this network meta-analysis, the use of probiotics is described as being well tolerated and safe in very low birth weight infants. Feeding intolerance and clinical sepsis were significantly reduced in the probiotic group compared to the control group. Interestingly, these studies also suggest that different outcomes may be influenced by feeding type (human milk vs. formula). This appears to further support the benefits of lactoferrin in combination with probiotics. Combining probiotics and lactoferrin may be a good idea for future research studies.

## 5. Limitations of the study

This network meta-analysis also has some limitations. This study only discussed the selection of probiotics for the prevention of NEC in very low birth weight infants, while the questions of the dosage, the timing of the intervention, and when to start the intervention remains unresolved. Most interventions were evaluated in only one or two trials, and only a few options were tested in four randomized controlled trials. Therefore, most probiotic interventions were evaluated in small experimental populations. In conclusion, the results of this study should be interpreted with caution, as the number of included trials was insufficient, so there was limited evidence for direct comparisons of some interventions, and further related studies are needed.

## 6. Conclusions

Our analysis suggests that Lactobacillus rhamnosus GG combined with bovine lactoferrin is the most effective and recommended regimen for preventing NEC in very low birth weight infants. However further studies are required to confirm this and also answer questions about probiotic dosage, timing and duration of therapy.

## Data Availability

The original contributions presented in the study are included in the article/**Supplementary Material**, further inquiries can be directed to the corresponding author/s.
