# Supplementary material for: Probiotics to prevent necrotizing enterocolitis in very low birth weight infants: A network meta-analysis
Source: Front Pediatr. 2023 Mar 6;11:1095368. doi: 10.3389/fped.2023.1095368 (PMC10025406; doi:10.3389/fped.2023.1095368)
Supplement: Supplementary file 1 [file Datasheet1.pdf]

Supplementary Material

Supplementary Table 1. Risk of bias for each included studies.

study	Randomization process	Deviations from intended interventions	Missing outcome date	Measurement of the outcome	Selection of the reported result	Overall Bias
Paolo Manzoni1	Low risk	Low risk	Low risk	Low risk	Low risk	Low risk
Gamze Demirel	Low risk	Low risk	Low risk	Some concerns	Some concerns	Some concerns
Ozge Serce	Low risk	Low risk	Low risk	Low risk	Low risk	Low risk
Kate Costeloe1	Low risk	Low risk	Low risk	Low risk	Low risk	Low risk
Sanjay Patole	Low risk	Some concerns	Low risk	Low risk	Low risk	Some concerns
Hung-Chih Lin	Low risk	Low risk	Low risk	Low risk	Low risk	Low risk
Mehmet Yekta Oncel	Low risk	Low risk	Low risk	Low risk	Low risk	Low risk
Susan E. Jacobs	Low risk	Low risk	Low risk	Low risk	Low risk	Low risk
Stephane Hays	Low risk	Low risk	Low risk	Low risk	Low risk	Low risk
Dilek Dilli	Low risk	Low risk	Low risk	Low risk	Low risk	Low risk
Kate Costeloe2	Low risk	Some concerns	Low risk	High risk	Low risk	High risk
İpek Güney-Varal	Low risk	Low risk	Low risk	Low risk	Low risk	Low risk
M Al-Hosni	Low risk	Some concerns	Low risk	Low risk	Low risk	Some concerns
Ozge Serce Pehlevan	Low risk	Low risk	Low risk	Low risk	Low risk	Low risk
W.A. Mihatsch	Low risk	Low risk	Low risk	Some concerns	Low risk	Some concerns
Iwona Sadowska-Krawczenko	Low risk	Low risk	Low risk	Low risk	Low risk	Low risk

Varaporn Saengtawesin	Low risk	Low risk	Low risk	Some concerns	Low risk	Some concerns
Carlo Dani	Low risk	Low risk	Low risk	Low risk	Low risk	Low risk
FN Sari	Low risk	Low risk	Low risk	Some concerns	High risk	High risk
Paolo Manzoni2	Low risk	Low risk	Low risk	Low risk	Low risk	Low risk
Erica L. Plummer2	Low risk	Low risk	Low risk	Some concerns	Low risk	Some concerns
T Havranek	Low risk	Low risk	Low risk	Low risk	Low risk	Low risk
Gayatri Athalye-Jape	Low risk	Some concerns	Low risk	Low risk	Low risk	Some concerns
P. Manzoni	Low risk	Low risk	Low risk	Low risk	Low risk	Low risk
Johanne E. Spreckels	Low risk	Some concerns	Low risk	Low risk	Low risk	Some concerns
Erik Wejryd	Low risk	Low risk	Low risk	Low risk	Low risk	Low risk
Nancy Patricia	Low risk	Low risk	Low risk	Low risk	Low risk	Low risk

Supplementary Table 2. Consistency test.

	Coef.	Std. Err.	z	P>z	[95% Conf.	Interval]
B VS CON	-2.175463	1.057283	-2.06	0.04	-4.2477	-0.1032268
C VS CON	0.3189079	0.5132835	0.62	0.534	-0.6871092	1.324925
D VS CON	-1.380876	0.8567938	-1.61	0.107	-3.060161	0.2984091
E VS CON	-0.6671799	0.6959577	-0.96	0.338	-2.031232	0.6968721
F VS CON	1.659472	0.8565744	1.94	0.053	-0.0193831	3.338327
G VS CON	0.5184438	0.5644797	0.92	0.358	-0.5879161	1.624804
H VS CON	0.4544081	0.6651001	0.68	0.494	-0.8491642	1.75798
I VS CON	0.9945171	0.3952117	2.52	0.012	0.2199164	1.769118
J VS CON	0.0950005	0.6366983	0.15	0.881	-1.152905	1.342906
K VS CON	0.7114073	0.9564879	0.74	0.457	-1.163274	2.586089
L VS CON	-1.549856	1.132498	-1.37	0.171	-3.769511	0.6697991
M VS CON	0.919724	0.5602625	1.64	0.101	-0.1783702	2.017818
N VS CON	0.9133327	0.4172567	2.19	0.029	0.0955246	1.731141
O VS CON	1.888066	1.577225	1.2	0.231	-1.203239	4.979371
P VS CON	-0.13642	0.6645145	-0.21	0.837	-1.438845	1.166005
Q VS CON	0.4599112	0.509019	0.9	0.366	-0.5377476	1.45757
R VS CON	-0.062444	1.237411	-0.05	0.96	-2.487726	2.362838
